# Creep Behavior of a Sn-Ag-Bi Pb-Free Solder

**DOI:** 10.3390/ma5112151

**Published:** 2012-11-02

**Authors:** Paul Vianco, Jerome Rejent, Mark Grazier, Alice Kilgo

**Affiliations:** Sandia National Laboratories, PO Box 5800, Albuquerque, NM 87185, USA; E-Mails: jarejen@sandia.gov (J.R.); jmgrazi@sandia.gov (M.G.); ackilgo@sandia.gov (A.K.)

**Keywords:** tin-silver-bismuth (Sn-Ag-Bi) solder, creep deformation, aging

## Abstract

Compression creep tests were performed on the ternary 91.84Sn-3.33Ag-4.83Bi (wt.%, abbreviated Sn-Ag-Bi) Pb-free alloy. The test temperatures were: −25 °C, 25 °C, 75 °C, 125 °C, and 160 °C (± 0.5 °C). Four loads were used at the two lowest temperatures and five at the higher temperatures. The specimens were tested in the as-fabricated condition or after having been subjected to one of two air aging conditions: 24 hours at either 125 °C or 150 °C. The strain-time curves exhibited frequent occurrences of negative creep and small-scale fluctuations, particularly at the slower strain rates, that were indicative of dynamic recrystallization (DRX) activity. The source of tertiary creep behavior at faster strain rates was likely to also be DRX rather than a damage accumulation mechanism. Overall, the strain-time curves did not display a consistent trend that could be directly attributed to the aging condition. The sinh law equation satisfactorily represented the minimum strain rate as a function of stress and temperature so as to investigate the deformation rate kinetics: dε/dt_min_ = Asinh^n^ (ασ) exp (−ΔH/RT). The values of α, n, and ΔH were in the following ranges (±95% confidence interval): α, 0.010–0.015 (±0.005 1/MPa); n, 2.2–3.1 (±0.5); and ΔH, 54–66 (±8 kJ/mol). The rate kinetics analysis indicated that short-circuit diffusion was a contributing mechanism to dislocation motion during creep. The rate kinetics analysis also determined that a minimum creep rate trend could not be developed between the as-fabricated versus aged conditions. This study showed that the elevated temperature aging treatments introduced multiple changes to the Sn-Ag-Bi microstructure that did not result in a simple loss (“softening”) of its mechanical strength.

## 1. Introduction

Predicting the reliability of solder interconnections, whether subjected to thermal mechanical fatigue (TMF), mechanical shock, or vibration, is depending to a greater extent upon computation modeling techniques. Interestingly, the capabilities of computer facilities and equipment have grown at such a rapid pace over the last 10–15 years that “CPU time” is not always the bottleneck to obtain timely, high-fidelity predictions. Rather, the limiting factor is now having access to the time-dependent (creep) and time-independent (stress-strain) mechanical properties of the materials that comprise the joint structure and, in particular, the solder. These properties are essential towards developing a unified creep-plasticity (UCP) constitutive equation that predicts deformation in the computational model [1,2]. In the study reported here, the creep behavior was evaluated for a Pb-free solder comprised of tin (Sn), silver (Ag), and bismuth (Bi). Although later variants of this material have included Cu additions, this effort examines the commercially-available, ternary alloy, 91.84Sn-3.33Ag-4.83Bi (wt.%, abbreviated Sn-Ag-Bi) [3]. 

The Sn-Ag-Bi alloy has a number of benefits. It has a relatively low solidus temperature of 212 °C. The Sn-Ag-Bi alloy exhibits exceptionally high strength values when compared to the other Pb-free compositions or the baseline eutectic Sn-Pb alloy. The Bi addition provides for excellent wetting-and-spreading behavior. A multiyear study examined the physical properties of the Sn-Ag-Bi solder as well as its performance in printed wiring assembly (PWA) interconnections [4,5,6,7,8,9,10]. Those studies examined the shear strength of ring-and-plug solder joints as well as the pull and shear strength of actual printed wiring assembly interconnections. Later studies explored the mechanical properties of this Sn-Ag-Bi composition as well as those of similar alloy contents. Kariya and Otsuka examined the isothermal fatigue of this solder [11] Shin and Yu investigated the creep behavior of the Sn-3.5Ag-xBi alloys (×, 2.5 and 7.5 wt.%) at 100 °C [12]. The single lap shear sample (solder joint thickness, 0.39 mm) resulted in shear stresses of 5–9 MPa. Strain rates ranged between approximately 6 × 10^−7^ s^−1^ and 5 × 10^−6^ s^−1^, resulting in stress exponents of 5.8 (2.5Bi) and 4.4 (7.5 Bi). 

The objective of the present study was to obtain a more complete compilation of the time-dependent deformation behavior of bulk Sn-Ag-Bi solder in order to support the development of a computational model for predicting the TMF of both electronic and structural solder joints. The effects of isothermal aging were also evaluated in this work. The methodologies used in the present study were similar to those used to evaluate the creep properties of the 95.5Sn-3.9Ag-0.6Cu solder (Sn-Ag-Cu) that is described in [13,14]. References [15,16,17] are excellent resources for the reader interested in obtaining more detailed information on the mechanical properties of these and other Pb-free solders.

## 2. Experimental Procedure

### 2.1. Test Samples 

The ternary alloy that was examined in this study had the composition, 91.84Sn-3.33Ag-4.83Bi (wt.%, abbreviated Sn-Ag-Bi). The compression test methodology was used for the creep tests. The test samples were created by first casting the material into cylinders. A density determination was made of each specimen to assure that significant voids were not present in the material. Then, the samples were machined to the nominal dimensions of 10 mm diameter and 19 mm length; the machining step also established parallelism between the end faces. These dimensions conformed to the “short length” ratio of 2.0 per the ASTM E9-89A specification [18]. A more detailed description of the sample fabrication equipment and procedures is available in [19]. The specimens were tested in the as-fabricated (as-cast) condition, that is, after casting and the machining operations. Additional samples were exposed to one of two aging temperature for 24 hours in air: 125 °C or 150 °C prior to the creep test.

### 2.2. Creep Testing 

Creep tests were performed on a servo-hydraulic frame using constant load control. The test temperatures were: −25 °C, 25 °C, 75 °C, 125 °C, and 160 °C (± 0.5 °C). The load values were chosen to provide nominal stresses in the range of 20%–80% of the estimated yield stress at that temperature (σ_y, T_). Four loads were evaluated at the temperatures of −25 °C and 25 °C while five loads were used at the three higher temperatures. The added loads were of lower values to capture subtle behaviors that could potentially take place under high temperatures and relatively slow strain rates. Duplicate specimens were tested under all stress and temperature combinations.

The duration of the creep tests was limited by either maximum strain reached by the sample, or a maximum time duration for the test. The strain limit was approximately 0.12. The resulting strain range is representative of that experienced by solder in joints that are undergoing TMF. In the event that the sample did not reach the strain limit, the test was halted after 1.73 × 10^5^ s (approximately two days). The reader is directed to [11] for additional details regarding the analyses that determined the values of true stress (σ), true strain (ε), and minimum strain rate (dε/dt_min_). A visual assessment of the curves was used to determine the presence of one, two, or three stages of creep. This method was determined to be as efficient as attempting to do it with a numerical scheme. Error terms that accompany the stresses and strain rates represent plus-or-minus one standard deviation over the time duration in which those respective data were collected and averaged together. Although standard convention would require the stress and strain rate values to be expressed as negative numbers (compression), they are reported here as positive values.

The deformation rate kinetics were determined from the minimum strain rate, dε/dt_min_, as a function of stress and temperature and expressed using the “sinh” law Equation (1):

(1) $$d\varepsilon /dt_{min}=A\;sinh^{p}\;(\alpha \sigma )\;exp\;\Delta H/RT).$$

The parameters in Equation (1) are: A, a constant (s^−1^); p, the sinh term exponent; α, the stress coefficient (MPa^−1^); σ, the applied stress (MPa); ΔH, the apparent activation energy; R, the universal gas constant (8.314 kJ/mol-K); and T, the temperature (K). The sinh law approach was preferred because it can represent a wider range of applied stresses and thus, avoid “power-law breakdown” that can occur when dε/dt_min_ is expressed using a power-law stress dependency. The parameters A, p, and ΔH were determined by a multivariable, linear regression analysis that was performed on the logarithm of Equation (1) that is represented by Equation (2) below:

(2) $$ln(d\varepsilon /dt_{min})=ln(A)+pln[sinh\;(\alpha \sigma )]-\Delta H/RT$$

The parameter, ln(dε/dt_min_), was the dependent variable while ln[sinh (ασ)] and 1/T were the independent variables. The regression analysis was performed for different values of the stress coefficient, α. The optimum value of α was determined to within ±0.005 by maximizing the square of the correlation coefficient, R^2^. The error terms on the sinh law parameters were expressed as the ± 95% confidence interval.

## 3. Results and Discussion

### 3.1. Strain-Time Curves 

The strain-time curves were analyzed according to sample condition and test temperature. These curves provide critical insight into microstructure changes that are not always readily visible in metallographic cross section, but nonetheless, have a significant role in the mechanical response of the material. The descriptions will be somewhat more detailed for the as-fabricated condition in order to establish the baseline behavior. That narrative will be followed by an analysis of results obtained from the aged samples. It is noted that the elastic strain was not subtracted from the total strain. Although the absence of this step precluded a quantitative comparison from being made between creep strains, qualitative comparisons could still be developed from the curves. Also, this omission did not interfere with an interpretation of the strain rate behavior. Lastly, the term “stress,” when alone, refers to the true stress. Nominal stress will be explicitly labeled as such in the discussion.

**Figure 1:**
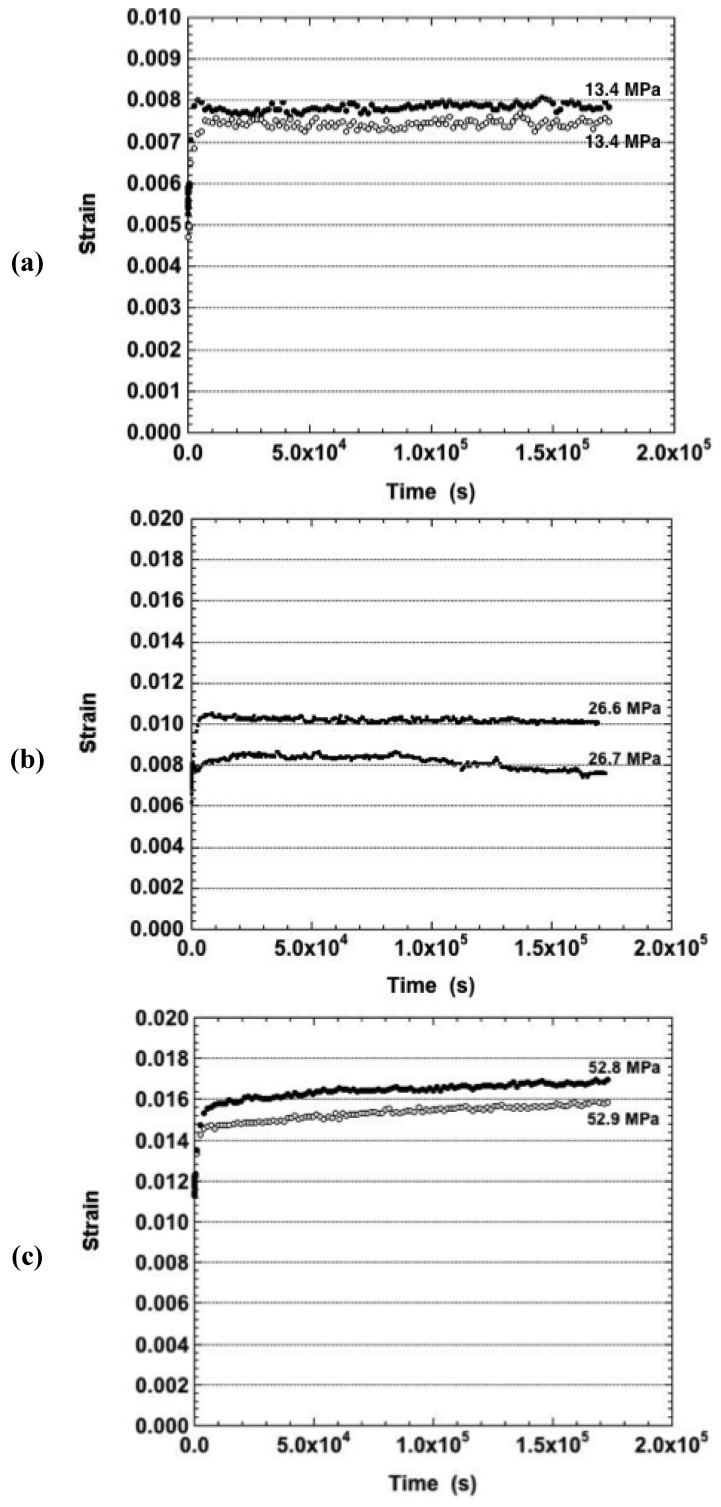
Duplicate strain-time curves of the Sn-Ag-Bi solder tested in the as-fabricated condition and at a temperature of −25 °C. The nominal stresses were: (**a**) 13.4 MPa; (**b**) 26.6 MPa; and (**c**) 52.9 MPa. Each curve is labeled with its corresponding true stress.

Shown in Figure 1a are the duplicate curves for the Sn-Ag-Bi samples tested in the as-fabricated condition; temperature of −25 °C; and stresses of 13.4 MPa. While one of the curves exhibited a small positive strain rate (2.2 × 10^−10^ s^−1^), the other curve showed negative creep (open circles). Negative creep could indicate of the simultaneous occurrence of mechano-chemical phenomenon in the material as suggested by Li [20]. The discussion in [20] refers to amorphous metals and specifically, microstructural disorder-to-order processes that occur in such materials leading to negative creep. The occurrence of such a mechano-chemical phenomenon in Sn-Ag-Bi, or similar consequence of DRX due to its associated changes to grain structure and defect density, are certainly possible, but would be only a hypothesis until validated by microstructural analysis.. Negative creep was also observed for both samples tested at −25 °C and the nominal stress of 26.6 MPa. This behavior is shown in Figure 1b and was more distinct in one sample than in the other sample. The stress of 39.8 MPa produced positive strain rates (3.1 × 10^−9^ s^−1^ and 5.0 × 10^−9^ s^−1^) as did also the stress of 52.8 MPa (4.0 × 10^−9^ s^−1^ and 4.2 × 10^−9^ s^−1^) as indicated in Figure 1c. The remainder of the strain-time curves representing the as-fabricated condition—regardless of stress or temperature—exhibited positive strain rates. 

**Figure 2:**
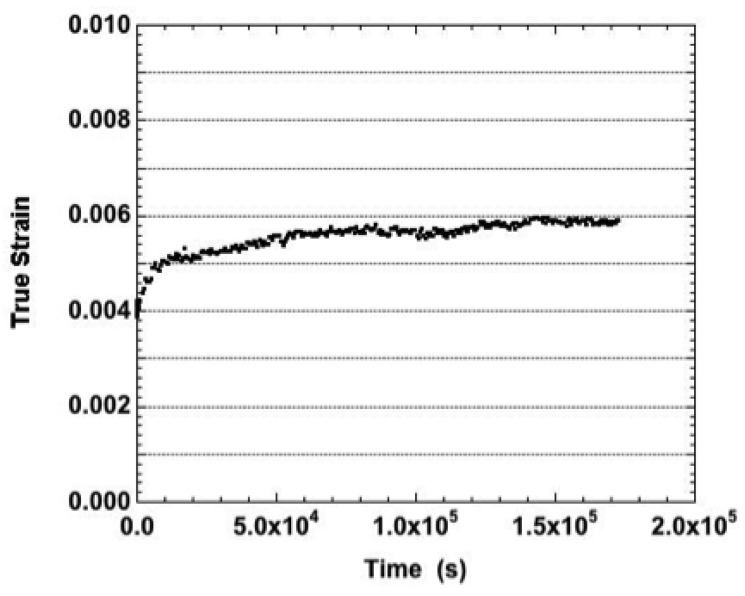
Strain-time curve of the Sn-Ag-Bi solder tested in the as-fabricated condition and at a temperature of 25 °C. The true stress was 11.5 MPa.

Several creep tests, when performed at 25 °C, exhibited fluctuations with amplitudes that exceeded the instrument error. The plot in Figure 2 (11.6 MPa) is representative of those fluctuations. Fluctuations were also observed on one of each of the curves obtained at 25 °C and nominal stresses of 23.2 MPa and 34.4 MPa. The fluctuations were absent from both 46.4 MPa strain-time curves. The fluctuations are also indicative of DRX and, moreover, portray the sub-class referred to as cyclic DRX. The cyclic softening and hardening correspond to the cycles of new grain initiation and grain growth, respectively, with respect to a grain boundary diffusion mechanism (discussed later). Lastly, all of the creep curves obtained at 25 °C exhibited primary creep; there was no component of steady-state (or secondary) creep in the strain-time data.

**Figure 3:**
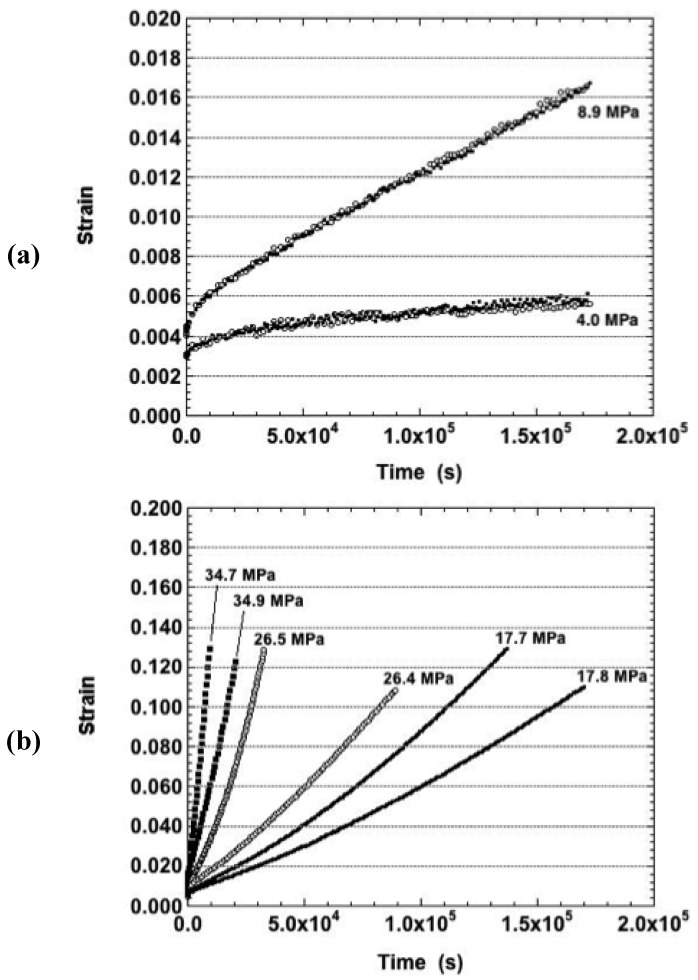
The strain-time curves of the Sn-Ag-Bi solder tested in the as-fabricated condition and at a temperature of 75 °C. The nominal stresses were: (**a**) 4.0 MPa and 9.0 MPa; and (**b**) 18.0, 27.0, and 36.0 MPa. The true stress values are shown on the plot.

The creep tests performed at 75 °C had, as the lowest two nominal stresses, 4.0 MPa and 9.0 MPa. The corresponding strain-time curves are shown in Figure 3a. In both cases, the duplicate curves overlapped one-another. (The true stresses were identical per the duplicate tests.) Shallow fluctuations were observed at 4.0 MPa that suggested a small contribution by cyclic DRX. Those fluctuations were absent at 8.9 MPa as well as from the strain-time curves of tests performed at all higher stresses.

The other stresses used at 75 °C had the nominal values of 18.0 MPa, 27.0 MPa, and 36.0 MPa. The corresponding curves are shown in Figure 3b and have been labeled with their true stress values. The creep rates increased with stress. All of the creep curves exhibited primary, secondary, and tertiary stages of creep. However, these curves exhibited by-and-large the tertiary stage. 

Traditionally, the increasing strain rate of tertiary creep indicates a ramp-up of microvoid coalescence and crack damage mechanisms that lead to final failure of the specimen. However, in light of potentially having DRX present during the creep of this material, it is proposed that tertiary creep may, in fact, be a consequence of that mechanism. Here is the proposed scenario: Assuming that a grain boundary diffusion-based process underlies the creep deformation, the creep rate would increase with decreasing grain size. Therefore, tertiary creep would indicate the grain initiation step in the DRX process, which when the grains are small and grain boundaries, most numerous. The result would be an acceleration of the strain rate. This mechanism will be discussed in detail, later on.

**Figure 4:**
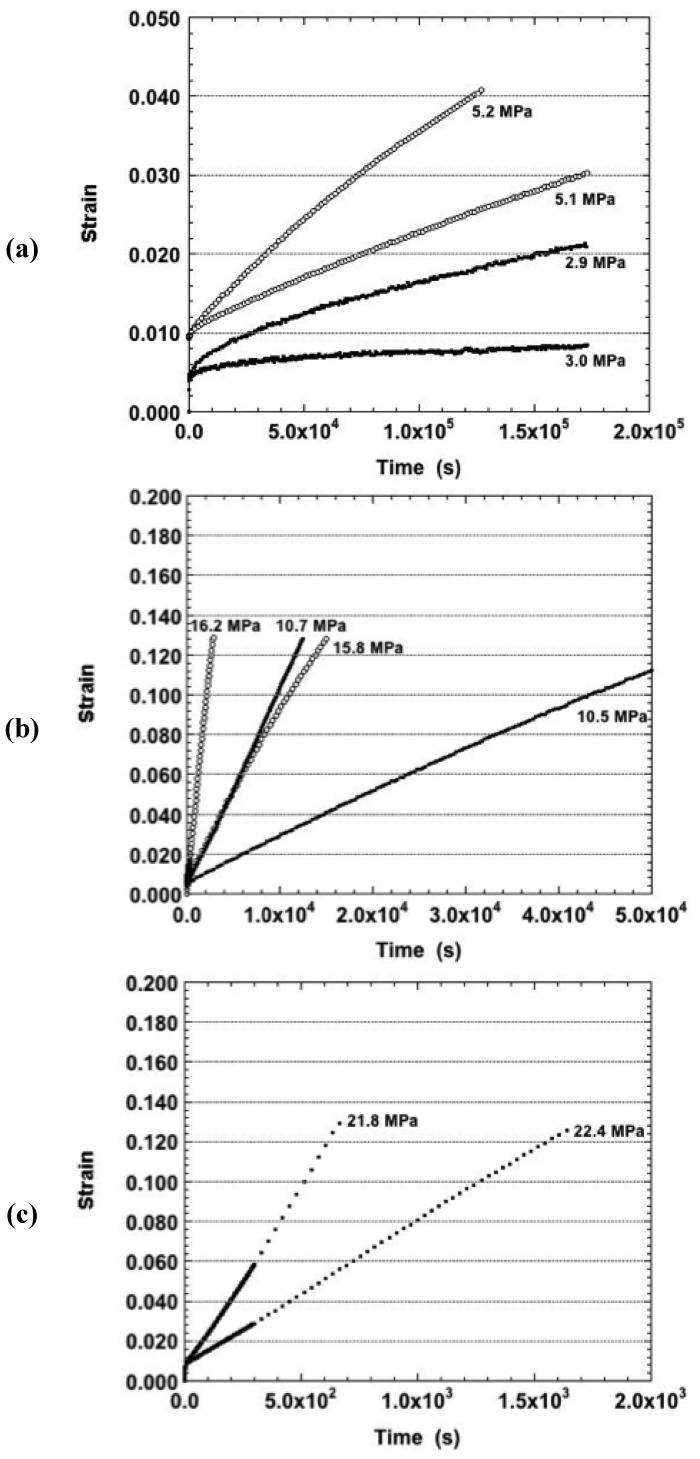
The strain-time curve of the Sn-Ag-Bi solder tested in the as-fabricated condition and at a temperature of 125 °C. The nominal stresses were: (**a**) 3.0 and 6.0 MPa; (**b**) 12.0 MPa and 18.0 MPa; and (**c**) 25.0 MPa.

The strain-time behavior was examined for the 125 °C test temperature. The creep curves are shown in Figure 4a for the true stresses of 2.9 MPa, 3.0 MPa, 5.1 MPa, and 5.2 MPa. The curves exhibited largely the primary stage and a small degree of secondary stage creep. The strain rates increased with stress and there were no indications of significant fluctuations. 

The curves obtained at true stresses of 10.5 MPa, 10.7 MPa, 15.8 MPa, and 16.2 MPa (125 °C) are shown in Figure 4b. When averaged together, the curves indicated an increase of strain rate with stress; but, individually, that trend was not entirely monotonic. This behavior suggests that there are significant microstructural differences in the starting Sn-Ag-Bi material because their effects persisted through the relatively long duration of the creep tests rather than impact only at the early stages of deformation. 

Figure 4c shows the plots obtained at 21.8 MPa and 22.4 MPa (125 °C). These tests were of relatively short durations (10,000 s = 2.7 hours) as the samples quickly reached the maximum strain. The curves exhibited a slight degree of tertiary behavior over, what was largely, secondary creep. Again, despite duplicate test conditions, the minimum strain rates were very different between the samples. 

**Figure 5:**
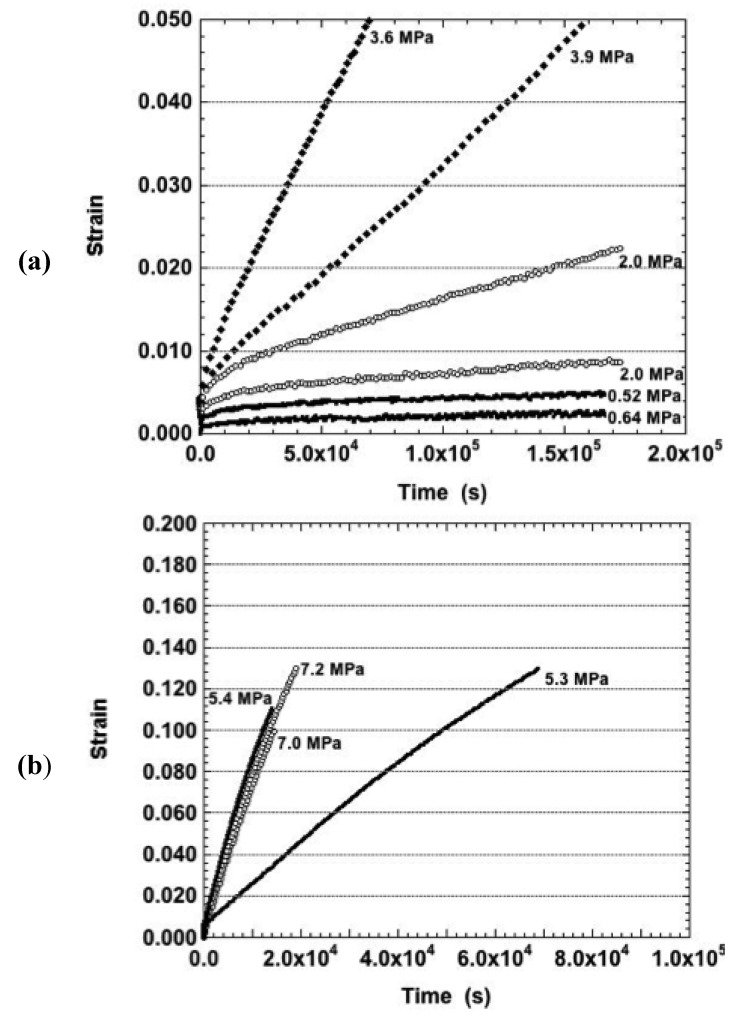
The strain-time curves of the Sn-Ag-Bi solder tested in the as-fabricated condition and at a temperature of 160 °C. The nominal stresses were: (**a**) 0.5–4.0 MPa; and (**b**) 6.0 MPa and 8.0 MPa.

The strain-time curves were examined that originated from (as-fabricated) samples tested at 160 °C. The duplicate strain-time responses are shown in Figure 5a for the nominal stresses of 0.5 MPa, 2.0 MPa, and 4.0 MPa. The lowest stress resulted in only primary creep. The other samples exhibited both primary and secondary stages except for the sample tested at 3.9 MPa, which also exhibited a small degree of tertiary creep. When averaged together, the curves demonstrated the expected increase of strain rate with increasing stress. But, a monotonic trend was not observed when considering all of the individual samples. The latter behavior was also observed at the higher stresses, the creep curves of which are shown in Figure 5b. Interestingly, those curves exhibited either primary creep, only, or a mixture of primary and a small degree of secondary creep. The absence of tertiary creep in Figure 5b, is further evidence that this behavior does not likely originate from a large-scale damage process. 

In summary, the strain-time curves were examined for the Sn-Ag-Bi solder when tested in the as-fabricated condition. Tests performed at −25 °C and 25 °C and lower stresses exhibited fluctuations indicative of DRX activity. The overall trend was primary creep. The DRX behavior was not observed at the higher stresses. The DRX fluctuations were recorded in only one other condition at higher temperatures: 75 °C, 4.0 MPa. At 75 °C, primary creep dominated the curves at stresses less than 18 MPa. At the higher stresses, primary, secondary and tertiary stage contributed to the deformation. The creep curves obtained at 125 °C and 160 °C were comprised of largely primary and secondary stages. Only a slight contribution was observed of the tertiary stage, and that occurred at the 125 °C test temperature. The lack of correlation between the presence of the tertiary stage versus strain rate, which determines the extent of strain deformation in the material, suggest that the source of the tertiary behavior is DRX and its related effects rather than microvoid coalescence and crack damage processes. 

**Figure 6:**
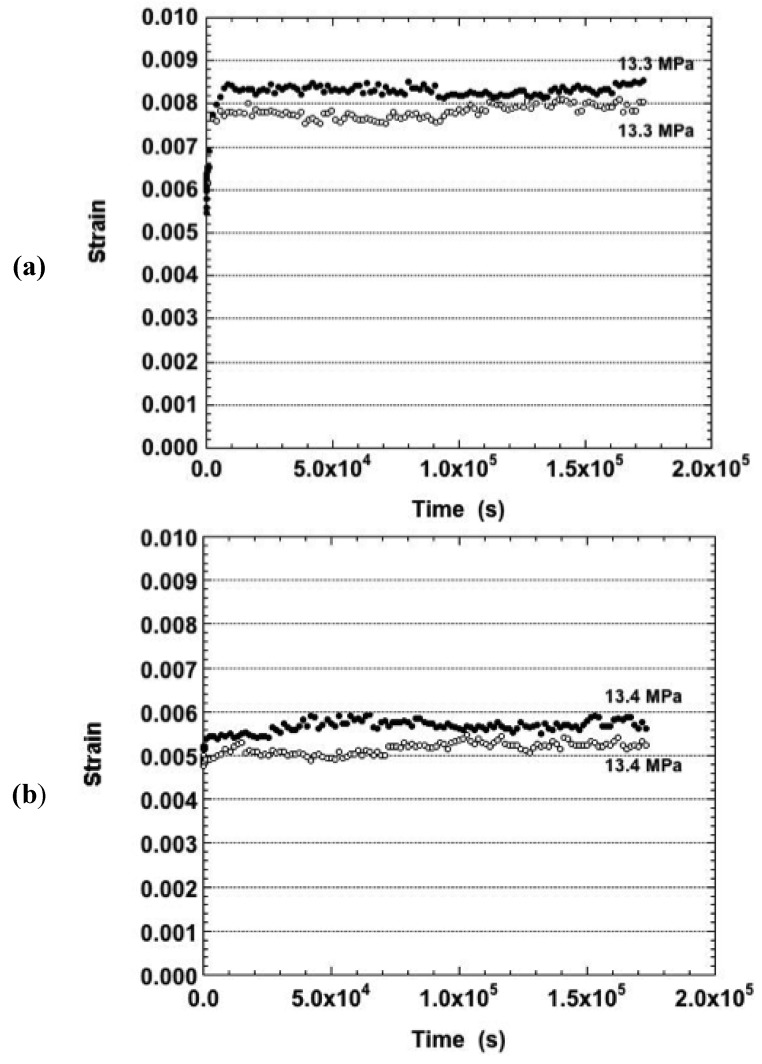
The duplicate strain-time curve of the Sn-Ag-Bi solder tested at −25 °C and nominal stress of 13.4 MPa and the two aging conditions: (**a**) 125 °C, 24 hours; and (**b**) 150 °C, 24 hours.

The strain-time curves were also analyzed that resulted from samples tested after aging for 24 hours at either 125 °C or 150 °C. Shown in Figure 6 are the duplicate creep curves obtained under the nominal stress of 13.4 MPa for the aging temperatures of (a): 125 °C; and (b): 150 °C. Amidst the fluctuations present in the curves, the long term strain rates were positive for both sample conditions. These curves can be compared directly to those of the as-fabricated condition in Figure 1a. Aside from different total strain values, all three sample conditions exhibited very similar strain-time behaviors.

Negative creep was observed at only one stress at −25 °C per each of the two aged conditions: 52.8 MPa (aged at 125 °C) and 26.6 MPa (aged at 150 °C). In the case of the as-fabricated samples tested at −25 °C, recall that negative creep was observed at 13.4 MPa and 26.7 MPa (−25 °C).

**Figure 7:**
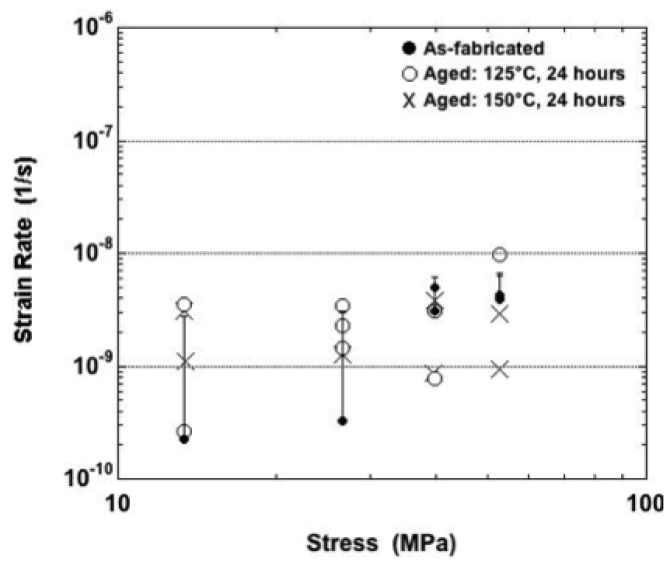
Minimum strain rate as a function of stress for samples tested at −25 °C and all three sample conditions.

The minimum strain-rate values are plotted in Figure 7 for tests performed at −25 °C. The one-half error bars (one standard deviation) were provided only for the as-fabricated condition because they were also representative of the two aging cases, thus avoiding cluttering of the plot. Only the as-fabricated samples exhibited an increase of strain rate with stress that had a statistical significance. No such trend was distinguishable for the aged samples, despite the very large stresses. Overall, there were no distinct trends of minimum strain rate as a function of the sample condition. 

The creep curves were examined that were obtained at a test temperature of 25 °C. Negative creep was observed in one of the two curves obtained at the nominal stress of 11.6 MPa. Otherwise, the three sample conditions exhibited very similar behaviors to within the variations documented between duplicate tests. Small fluctuations, which were indicative of mild DRX, were superimposed over generally positive strain rates. Those fluctuations rapidly disappeared with increasing stress. The strain-time curves were dominated by primary creep for all three conditions, except at the highest nominal stress (46.4 MPa) at which secondary creep appeared in the plots. 

**Figure 8:**
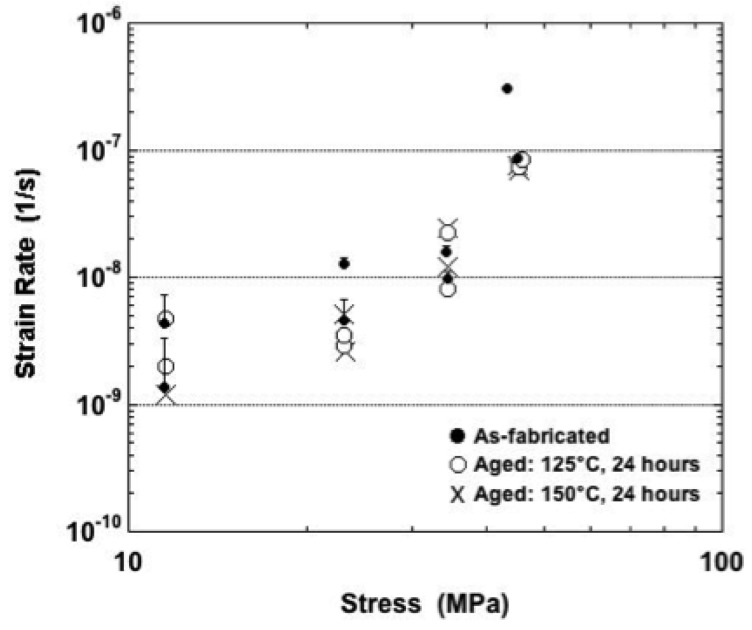
Minimum strain rate as a function of stress for samples tested at 25 °C and all three sample conditions.

The minimum strain rate data have been plotted in Figure 8 that were obtained from tests performed at 25 °C. The values were similar at the two lowest stresses, 11.6 MPa and 23.2 MPa for all aging conditions. Overall, the minimum strain rate did not exhibit a consistent, statistically significant dependency on aging condition across the range of stress values. The strain rate increased with stress, slowly at the lower stresses and then more rapidly at the higher stresses.

**Figure 9:**
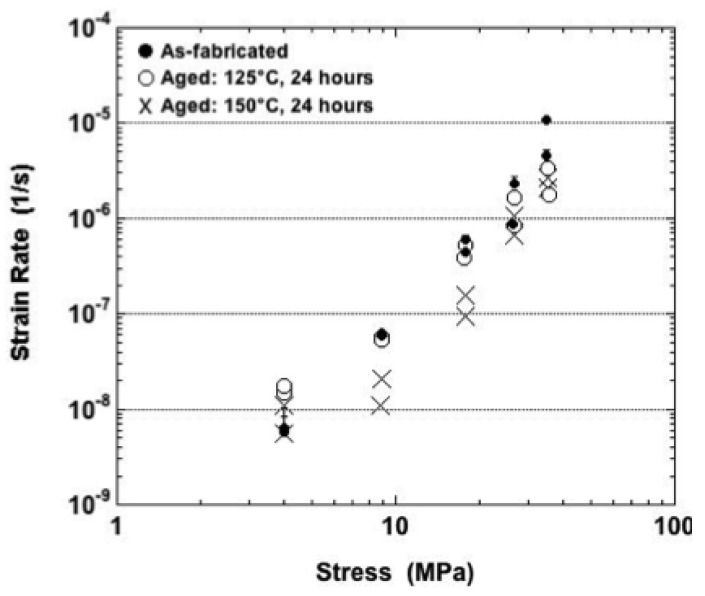
Strain rate as a function of stress for samples tested at 75 °C and all three sample conditions.

The 75 °C test temperature marked changes to the behaviors between the three sample conditions. At the lowest nominal stress of 4.0 MPa, the Sn-Ag-Bi material performed very similarly between the three aging conditions. There were small fluctuations on top of a net positive strain rate of primary creep. When the nominal stress was increased to 9.0 MPa, the small-scale fluctuations disappeared and the strain-time plots were comprised of both primary and secondary stages. The most noticeable difference was that the 150 °C aging treatment caused a significant reduction in minimum strain rate and, as such, creep strain. This observation is illustrated in Figure 9, which shows minimum strain rate as a function of stress for each of the three aging conditions (75 °C). 

**Figure 10:**
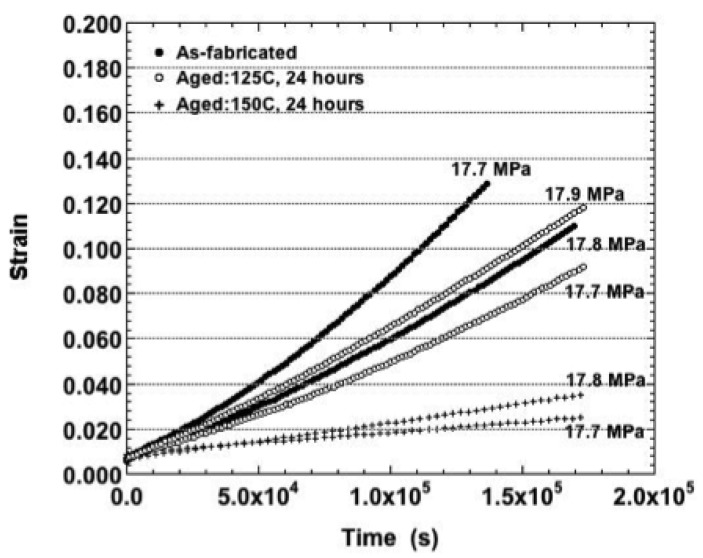
The duplicate strain-time curves of the Sn-Ag-Bi solder tested at 75 °C and nominal stress of 18.0 MPa and the three aging conditions: as-fabricated, 125 °C, 24 hours and 150 °C, 24 hours.

The effect of the 150 °C aging treatment was further accentuated under the 18.0 MPa nominal stress (75 °C). The corresponding strain-time curves are shown in Figure 10. Lower creep strains and strain rates (Figure 9) were observed at this nominal stress for the samples. Those curves experienced only a small degree of tertiary creep as compared to the other curves that were almost entirely tertiary in nature. 

At a nominal stress of 27.0 MPa, the strain-time curves of the aged samples all fell between the duplicate, as-fabricated condition curves that were shown in Figure 3b. In all cases, the maximum strain (0.12) was met or nearly so, and the curves were largely of the tertiary stage. Similar observations pertained to the highest nominal stress of 36.0 MPa. Referring to Figure 9, in the case of these two highest stresses, the minimum strain rate did not exhibit a significant trend as a function of aging treatment.

When the test temperature was raised to 125 °C, only the nominal stress of 3.0 MPa caused any distinction between the three sample conditions. Because the maximum strain had not been achieved in these tests, it was possible to observe that the 150 °C aging condition resulted in a significantly lower total creep strain than was observed for the other two conditions. At the higher stresses, it appeared that the samples aged at 150 °C also exhibited a slightly lesser degree of variability between duplicate tests than did the other sample conditions. The strain-time curves progressed from largely primary creep (3.0 and 6.0 MPa) to a mixture of all three stages (12.0 and 18.0 MPa) and lastly, secondary creep with a small tertiary contribution (25.0 MPa). Any small-scale fluctuations, which have been attributed to DRX, were absent from all of the plots. 

**Figure 11:**
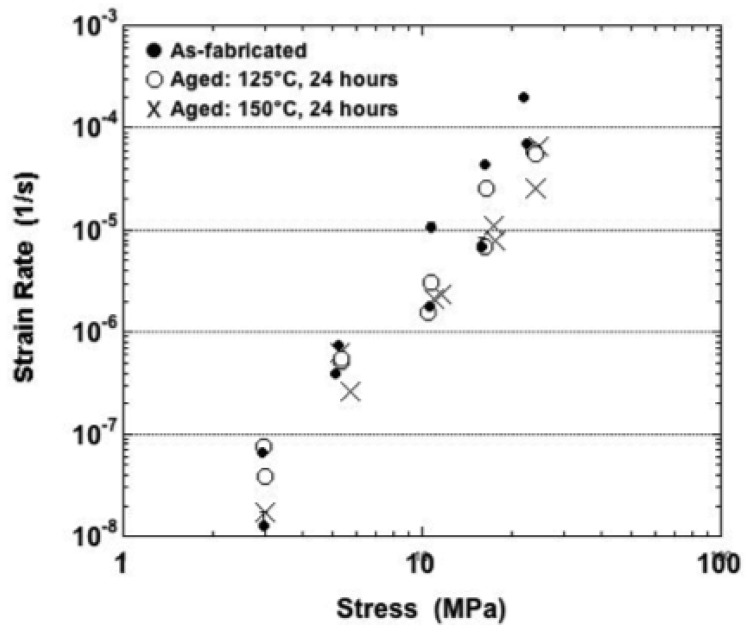
Minimum strain rate as a function of stress for samples tested at 125 °C and all three sample conditions.

Shown in Figure 11 are the minimum strain rates plotted as a function of stress (125 °C). The strain rate increased significantly with stress. Given the relatively small error bars associated with these data, it appears that more often than not, the as-fabricated condition resulted in slightly faster, minimum strain rate values while aging at 150 °C (24 hours) tended to cause lower minimum strain rates. 

The final test temperature was 160 °C. At the lowest nominal stress of 0.5 MPa, the creep curves were very similar between all three sample conditions to within the observed variations. The duplicate curves were more alike to one-another after the 150 °C aging treatment. The strain-time responses exhibited small fluctuations superimposed on a general trend of primary creep. 

**Figure 12:**
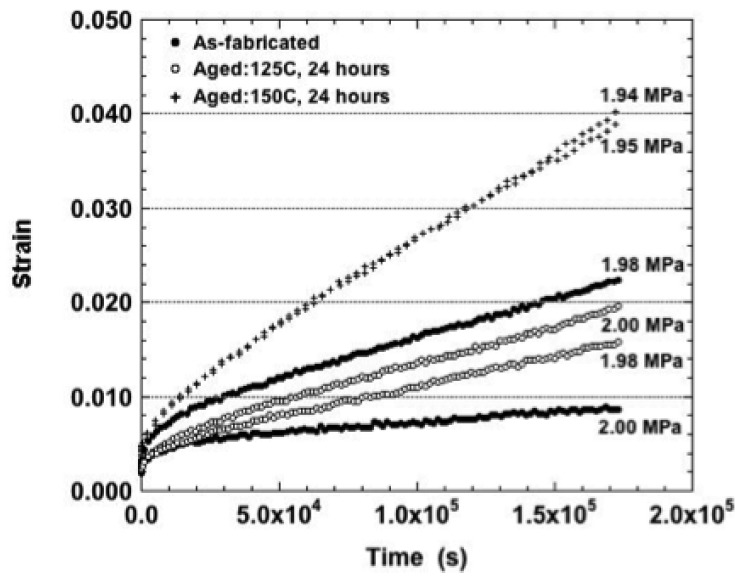
The duplicate strain-time curve of the Sn-Ag-Bi solder tested at 160 °C and nominal stress of 2.0 MPa and the three aging conditions.

The effects of the aging treatment first appeared at the 2.0 MPa nominal stress. The strain-time plots are shown in Figure 12 for each of the sample aging conditions. The curves were comprised of both primary and secondary stages; tertiary creep was absent from all plots. There was an absence of small fluctuations beyond the measurement error. The 150 °C aging treatment caused a significant increase in strain rate (and creep strain). Also, the duplicate curves were nearly on top of one-another as compared to the larger variability observed for the other two sample conditions.

The nominal stresses of 4.0 MPa and 6.0 MPa (160 °C) resulted in strain-time curves that were not particularly distinguishable between the three conditions. The strains reached the maximum limit in all cases and small fluctuations remained absent from the plots. The curves, themselves, were comprised of a “shallow” primary stage and dominating secondary stage. There was a greater variability between duplicate curves at 6.0 MPa than was observed for the plots representing 4.0 MPa. 

**Figure 13:**
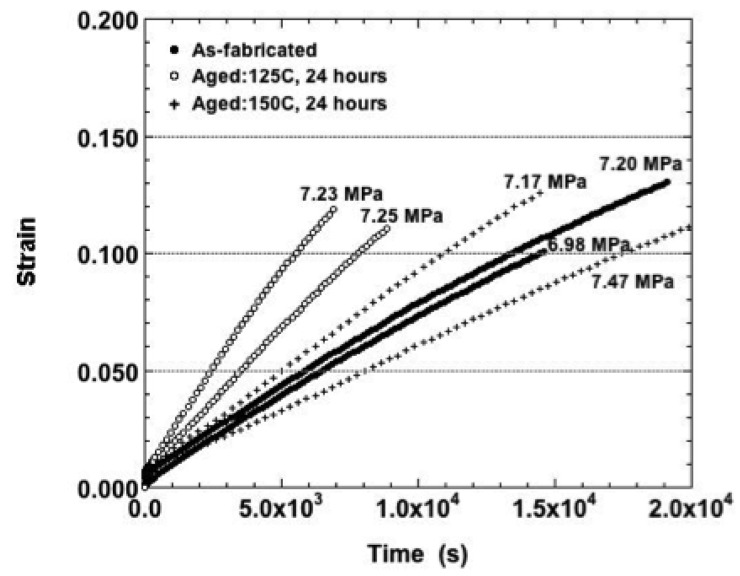
The duplicate strain-time curve of the Sn-Ag-Bi solder tested at 160 °C and nominal stress of 8.0 MPa and the three aging conditions: as-fabricated, 125 °C, 24 hours and 150 °C, 24 hours.

The highest nominal stress used at 160 °C was 8.0 MPa. Those strain-time curves appear in Figure 13. In this case, it was the samples aged at 125 °C that exhibited the faster strain rates. (The creep strains had reached the test limit.) It is noted that all of the curves exhibited a primary creep stage of modest slope that approached a secondary stage. Tertiary behavior was absent in all cases.

**Figure 14:**
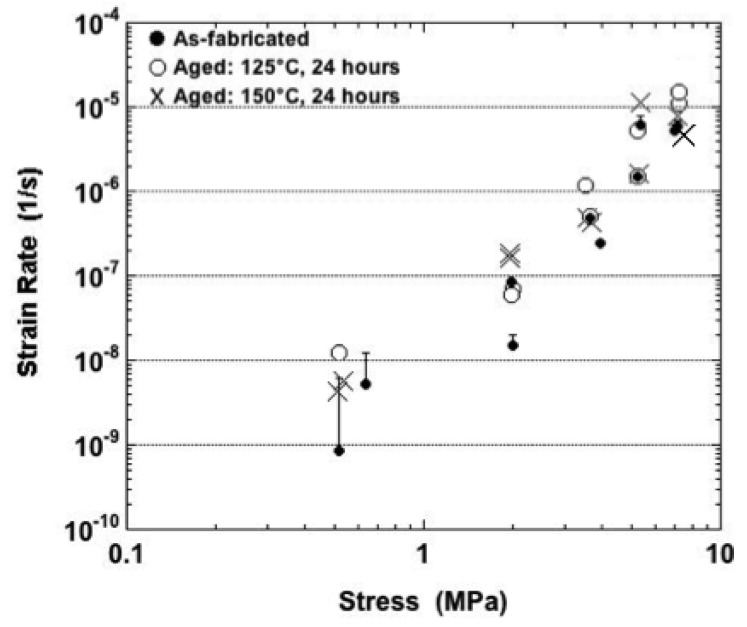
Minimum strain rate as a function of stress for samples tested at 160 °C and all three sample conditions.

The minimum strain rate data are plotted as a function of stress for the 160 °C tests temperature in Figure 14. The strain rate values exhibited a significant stress dependence. After taking into account the error bars, it appeared that the aged samples had slightly faster strain rates than did the as-fabricated samples at nominal stresses of 4.0 MPa and less.

An interim summary was made of the observations compiled from the empirical strain-time and strain rate data of the Sn-Ag-Bi solder. Listed below are the high-level trends as a function of stress, temperature, and aging treatment:

(a) The small-scale fluctuations and isolated occurrences of negative creep indicated that DRX processes were active. These phenomena were most obvious under those test conditions that gave rise to slower strain rates.
(b) The tests performed at 75 °C exhibited a wide range of curve shapes as a function of increasing stress. The curves started out with largely primary creep at low stresses and then transitioned to a mixture of primary-plus-secondary stages. At the highest stresses, the strain-time curves exhibited largely the tertiary creep behavior.
(c) The tertiary creep stage contributed very little to the strain-time curves at 125 °C and was absent from those obtained at 160 °C, despite the rapid strain rates and large creep strains.
(d) The tertiary strain-time behavior was not consistent with a traditional damage accumulation process. Rather, it originated from DRX or was an effect of DRX (e.g., grain size change). However, other mechanisms, such as precipitation hardening/softening, cannot be completely ruled out.
(e) Overall, the strain-time curves did not display a consistent trend that could be attributed to the aging condition.. This behavior is not unexpected because the elevated temperatures provide an opportunity for microstructural phenomenon such as DRX or precipitation hardening/softening to take place simultaneously with the deformation.
(f) The individual, minimum strain rate data corroborated the above observations. Further use is made of the strain rate data in the following section that discusses deformation rate kinetics.

### 3.2. Deformation Rate Kinetics 

The creep deformation rate kinetics were calculated from the minimum strain rate, stress, and temperature as expressed in equation (1). The equations for each of the three aging conditions are shown below:

As-fabricated:

(3) $$d\varepsilon /dt_{min}=3.225\times 10^{5}\;sinh^{3.1\;\pm \;0.4}\;(0.015\sigma )\;exp\;(-66\pm 7/RT)$$

Aged: 125 °C, 24 hours:

(4) $$d\varepsilon /dt_{min}=3.150\times 10^{3}\;sinh^{2.2\;\pm \;0.5}\;(0.010\sigma )\;exp\;(-54\pm 7/RT)$$

Aged: 150 °C, 24 hours:

(5) $$d\varepsilon /dt_{min}=4.803\times 10^{3}\;sinh^{2.2\;\pm \;0.5}\;(0.010\sigma )\;exp\;(-56\pm 8/RT)$$

The confidence intervals on the coefficient, A, were ±20, ±20, and ±30, respectively. These values are very small, relative to the mean values in the equations, because the regression analyses delivered the logarithms of the mean and standard errors, which were then converted to their nominal values. Lastly, the respective R^2^ values are 0.906, 0.865, and 0.840. These values indicate that the sinh law expression provided a satisfactory fit to the respective experimental data sets. 

Observations are made with respect to the values of stress coefficient, α, (in the sinh argument); the sinh term exponent, n; and the apparent activation energy, ΔH. The value of α is relatively small, which tends to de-amplify the effect of stress on the minimum strain rate. A similar effect can be attributed to n, that is, a smaller value of n causes the minimum strain rate to be less sensitive to stress. Comparing the three equations above, the minimum strain rate was most sensitive to stress when Sn-Ag-Bi was tested in the as-fabricated condition. The reduced stress sensitivities that were observed for the samples of the two aged conditions, were identical to one-another. 

It is possible to draw a correlation between the value of n and possible deformation mechanisms because the product of ασ is less than 0.8 so that the sinh law representation can be approximated by the common power law expression, σ^n^. Given this similarity, it is possible to exploit the following established correlations between n and specific deformation mechanisms:

- n = 1, diffusion mechanisms (Coble or Nabarro-Herring creep);
- n > 3, dislocation mechanisms (glide, climb, or climb-assisted glide).

Besides supporting their own respective deformation processes, diffusion, dislocation or both mechanisms in combination, can be rate-controlling for the grain boundary sliding deformation process. Lee and Stone demonstrated that the value of n can be in the range of 1 < n < 2.5 for grain boundary sliding in Pb-Sn alloy [21]. However, the value is highly dependent on grain size as well as other microstructural features and can significantly exceed this range in other materials [22]. 

Based on these two limiting mechanisms indicated by n, it appears that Sn-Ag-Bi creep is controlled by dislocation activity (glide or climb) when in the as-fabricated condition. This trend would also corroborate with the higher value of α, which indicates a greater effect by stress that is expected when dislocation activity is present. However, the magnitude of n is near the lower limit considered indicative of dislocation activity, implying that a diffusion-based mechanism may have a contributing role in the creep behavior. After either aging treatment, the still lower value of n implies that a diffusion mechanism has an increased role in creep behavior of Sn-Ag-Bi. A microstructure analysis would be required to confirm the process actually responsible for the creep deformation (e.g., simple dislocation motion or the more complex grain boundary sliding)

The third parameter in the sinh law Equations (3–5) is the apparent activation energy, ΔH. Per the 95% confidence interval, ΔH was statistically the same between all three sample conditions. The values are indicative of a short-circuit diffusion process rather than bulk diffusion (which would have ΔH values of 90–110 kJ/mol for these materials). Given that the values of n, above indicated the likelihood that diffusion contributed to the creep of Sn-Ag-Bi, the ΔH values would certainly support that observation. The diffusion-based mechanism would support a Coble creep process, which is based on grain boundaries providing the short-circuit path, as opposed to Nabarro-Herring creep, which is controlled by bulk diffusion.

**Figure 15:**
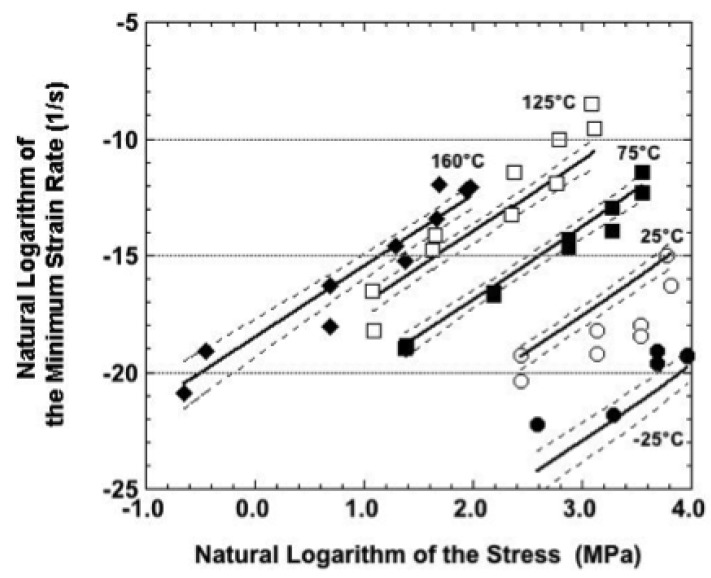
Natural logarithm of the strain rate as a function of that of the stress for samples in the as-fabricated condition. The symbols are the experimental data. The solid lines are the best-fit to those data predicted by sinh law representation. The dashed lines are the 95% confidence intervals to that prediction.

A comparison was made between the experimental creep data and the sinh law predictions. The discussion is categorized according to sample condition. Shown in Figure 15 is a plot of the natural logarithm of the minimum strain rate (dε/dt_min_) as a function of the natural logarithm of the applied stress, σ, for the as-fabricated condition. The symbols are the experimental data. The solid lines are the predicted trends generated according to Equation 3. The accompanying dashed lines represent the 95% confidence intervals. The sinh law slightly under-predicted the strain rates at −25 °C. This discrepancy was not unexpected, given the relatively small strains and strain rates experienced by the Sn-Ag-Bi alloy at this temperature. 

On the other hand, the sinh law model significantly over-predicted the strain rates observed at 25 °C. Although negative creep was not observed in these samples, moderate fluctuations, which are indicative of cyclic DRX, were superimposed on the strain-time curves at all but the highest nominal stress. It was concluded that the grain growth stage of DRX, which increases grain size, was responsible for the lower-than-expected experimental strain rate.

Reasonably good fits were observed between the sinh law predictions and experimental data at the other three test temperatures: 75 °C, 125 °C, and 160 °C. Although the tertiary stage behavior became a significant contributor to the overall creep response at 75 °C, the minimum creep rate preceded the tertiary stage. As such, the DRX mechanism, which was proposed as the source of the tertiary behavior, would not have yet affected the creep behavior. At 125 °C and 160 °C, the experimental strain rates observed at the highest nominal stress tended to be above the sinh law trend line. Such a behavior would suggest the occurrence of a “power law breakdown-like event,” but one of such a magnitude as to exceed the ability of even the sinh law to represent it. Mechanistically, the breakdown event would have indicated the loss of the diffusion contribution to one entirely controlled by dislocation motion (glide, climb, a combination of the two mechanisms, or grain boundary sliding) under the higher stresses. 

**Figure 16:**
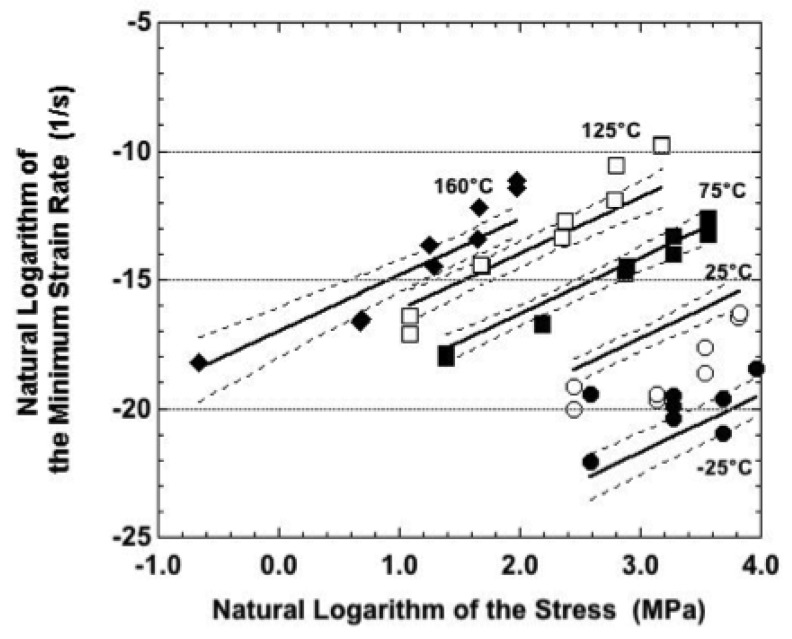
Natural logarithm of the strain rate as a function of that of the stress for samples that were aged at 125 °C for 24 hours prior to testing. The symbols are the experimental data. The solid lines are the best-fit to those data predicted by sinh law representation. The dashed lines are the 95% confidence intervals to that prediction.

Shown in Figure 16 are the experimental data and sinh law predictions made by Equation (4) for samples aged at 125 °C for 24 hours. The sinh law fit slightly under-predicted the −25 °C response and over-predicted the strain rates at 25 °C as was also the case for the as-fabricated condition (Figure 15). Certainly, some of the discrepancy can be attributed to the non-linearity (or “knee”) between the logarithms of strain rate and increasing stress; it is particularly evident in the 25 °C test data. Because small fluctuations were observed on the strain-time curves, the grain-growth step in cyclic DRX would be a contributing factor here as was suggested for the as-fabricated samples. 

In general, there was very good correlation between the experimental data and sinh law predictions for the three higher temperatures: 75 °C, 125 °C, and 160 °C. The fit was particularly good at 75 °C. At 125 °C and 160 °C, the empirical strain rates were above the sinh law prediction at the highest respective stress values, suggesting a possible breakdown event in the rate-controlling creep mechanism.

**Figure 17:**
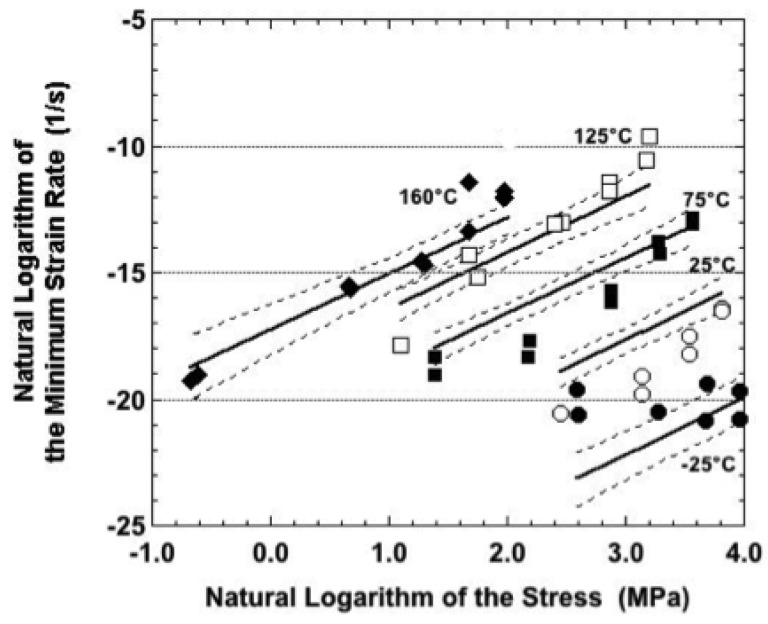
Natural logarithm of the strain rate as a function of that of the stress for samples that were aged at 150 °C for 24 hours prior to testing. The symbols are the experimental data. The solid lines are the best-fit to those data predicted by sinh law representation. The dashed lines are the 95% confidence intervals to that prediction.

The minimum strain rate data and sinh law predictions of Equation (5) are plotted in Figure 17 for samples aged at 150 °C for 24 hours. The R^2^ value indicated the poorest fit, although still satisfactory, between the sinh law and the experimental data. The reduced correlation began with −25 °C case. At the lowest nominal stress of 13.4 MPa, the duplicate strain rates were higher than was predicted by the sinh law equation. The two strain-time curves were shown in Figure 6b. Given the moderate fluctuations, it is certainly feasible that the faster strain rate was due to the cyclic DRX process, but this time, under the grain initiation step. The initiation of new grains increases the number of grain boundaries that, in turn, increases the number of diffusion paths supporting a Coble creep process. The correlation improved at the higher stresses.

As was the case in Figure 15 and Figure 16, the sinh law model over-predicted the strain rates at 25 °C compared to the experimental data. The “knee” in the test data is very prominent; the best fit occurred at the highest stresses where the data were within the 95% confidence interval. 

Unlike the previous two samples conditions, the lack of correlation between the sinh law fit and empirical data persisted through the higher test temperatures. At 75 °C, a “knee” in the empirical data resulted in the latter having a lower-than-predicted minimum strain rate at 9.0 MPa and 18.0 MPa nominal stresses. At 125 °C and 160 °C test temperatures, the best fit between the test results and sinh law predictions occurred at the mid-range stresses. The sinh law slightly over-predicted the strain rate at the lowest stresses. But, more so, the sinh law under-predicted the strain rates at the highest stresses. This trend was similar to the earlier observations that the Sn-Ag-Bi alloy was susceptible to a breakdown event when creep tested under the higher stress values. Although the breakdown was observed under all three sample conditions, it was most pronounced in samples that were aged at 150 °C. 

**Figure 18:**
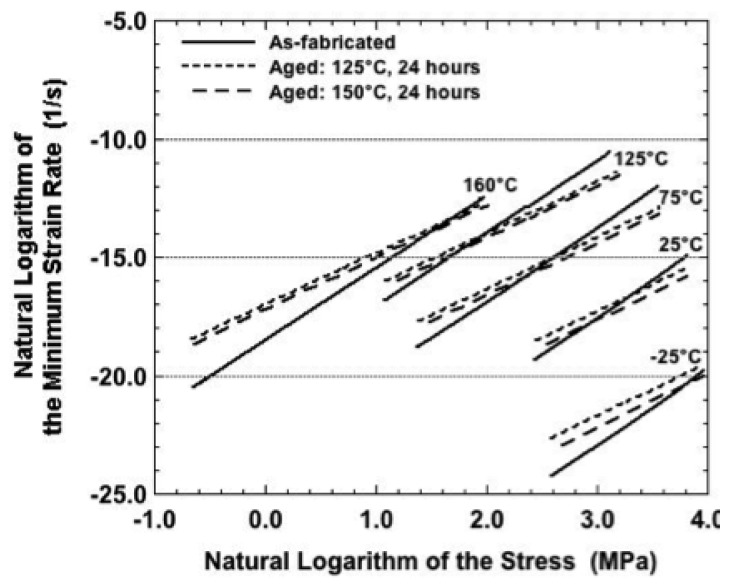
Natural logarithm of the minimum strain rate as a function of that of the stress as predicted by the sinh law equations for each of the three sample conditions.

Recall that the plots in Figure 7, Figure 8, Figure 9, Figure 11 and Figure 14 compared experimental, minimum strain rate values between the three sample conditions according to each of the test temperatures. A similar comparison was made of the predictions provided by the sinh law Equations (3–5) in Figure 18. In this case, the trend lines were combined on a single plot that included test temperature and sample condition dependencies. (The confidence intervals were left off the plots for clarity.) The following observations were made from Figure 18:

(a) The slope of the as-fabricated condition is steeper than those of the aging treatments, indicating a greater sensitivity of minimum strain rate to stress. The slopes were nearly identical between the two aging conditions. There are two scenarios to explain this trend. The first scenario is that the aging treatments add obstacles to the motion of existing dislocations. Such a case would prevail if the aging treatments caused solute precipitation. The second scenario would have the aging treatments annihilate dislocations. Thus, the deformation rate would be limited after the aging treatment until there could be an increase in the dislocation density. Certainly, it is possible that both scenarios contributed to the observed trend.
(b) It was not possible to develop a consistent trend of minimum strain rates between as-fabricated versus aged sample conditions because the traces crossed over one-another at different stresses, depending upon the temperature. Over the range of stresses used in this study, that inconsistency occurred to the least extent at −25 °C and 160 °C. In those instances, the as-fabricated condition was predicted to have a lower strain rate than are predicted for the two aging conditions.
(c) Comparing the two aging conditions, samples annealed at 150 °C (24 hours) caused a lower, minimum strain rate than was observed for samples aged at 125 °C. This result further supports the inference made above with respect to Figure 15, Figure 16 and Figure 17: The aging treatments cause changes to the Sn-Ag-Bi microstructure other simply decreasing its strength due to recovery and/or static recrystallization mechanisms. Alternative processes include the DRX concept described in this analysis as well as the roles of solute precipitation and changes to the dislocation density (dynamic recovery).

It is instructive to summarize the observations compiled from Figure 15, Figure 16 and Figure 17. First of all, across all three sample conditions, the sinh law equations consistently under-predicted the strain rate at −25 °C and over-predicted the strain rate at 25 °C. This discrepancy further substantiates the earlier summation that other microstructural processes—most likely DRX mechanisms, but certainly not excluding other possibilities such as dynamic recovery and solute precipitation—were occurring simultaneously during creep deformation.

Secondly, the correlation between the empirical data and the sinh law equations was improved at the higher test temperatures of 75 °C, 125 °C, and 160 °C. The fit was deemed to be very good for the as-fabricated condition as well as for samples that had been aged at 125 °C for 24 hours, perhaps slightly better in the latter case for these test temperatures. However, the correlation decreased for samples aged at 150 °C. In the latter case, the samples tested at 75 °C behaved more like those tested at 25 °C, showing a “knee” in the data. The 125 °C and 160 °C data were better correlated to the sinh law predictions, but largely so at the middle stresses. Otherwise, the correlation deteriorated at the lowest and highest stresses.. 

Third, the data suggest that a measure of stabilization can be introduced into the Sn-Ag-Bi microstructure using the 125 °C, 24 hour aging treatment. Although the 150 °C aging temperature improved the reproducibility of strain-time curves at a given set of test parameters, it is clear that, in terms of secondary creep strain rate, this aging condition initiated other microstructural changes that lessened the predictability of the creep behavior using the sinh law representation.

## 4. Conclusions

1. Compression creep tests were performed on the ternary alloy 91.84Sn-3.33Ag-4.83Bi (wt.%, abbreviated Sn-Ag-Bi) Pb-free solder. The test temperatures were: −25 °C, 25 °C, 75 °C, 125 °C, and 160 °C (±0.5 °C). Four loads were used at the two lowest temperatures and five loads at the three remaining temperatures. The specimens were tested in the as-fabricated condition or after being subjected to one of two aging conditions: 24 hours at either 125 °C or 150 °C. All aging treatments were performed in air.
2. The strain-time curves provided evidence by means of negative creep and small-scale fluctuations, the latter suggesting that DRX was active during creep. The evidence was most obvious at the slower strain rates.
3. The tertiary strain-time behavior, which was observed usually at faster strain rates, was not the consequence of a traditional damage accumulation process. Rather, it was proposed that it originated from DRX. However, it is recognized that other mechanism such as precipitation hardening/softening have not been completely ruled out in the absence of a microstructural analysis.
4. Overall, the strain-time curves did not display a consistent trend that could be attributed to the aging condition.
5. The sinh law equation, dε/dt_min_= Asinh^n^ (ασ) exp (−ΔH/RT), was used to analyze the creep rate kinetics. The values of α, n, and ΔH had these ranges across sample aging conditions: α, (0.010–0.015) ± 0.005 MPa^−1^; n, (2.2–3.1) ± 0.5; and ΔH, (54–66) ± 8 kJ/mol. The rate kinetics parameters indicated that short-circuit diffusion was a contributing mechanism to that of dislocation motion in the creep of this alloy.
6. The sinh law representations did not show a consistent trend of between minimum creep rate between the as-fabricated versus aged conditions. However, there is evidence that the 125 °C, 24 hour aging treatment provided a slightly greater degree of stabilization to the minimum creep rate behavior of the Sn-Ag-Bi alloy.
7. Discrepancies between the sinh law prediction and empirical test data were observed at the lowest temperatures, −25 °C and 25 °C, which were likely due to the effects of DRX. A breakdown event was observed at the highest temperatures and highest stresses.
